# SNPs detection by eBWT positional clustering

**DOI:** 10.1186/s13015-019-0137-8

**Published:** 2019-02-06

**Authors:** Nicola Prezza, Nadia Pisanti, Marinella Sciortino, Giovanna Rosone

**Affiliations:** 1 Dipartimento di Informatica, University of Pisa, Pisa, Italy; 2 Dipartimento di Matematica e Informatica, University of Palermo, Palermo, Italy; 3 ERABLE Team, INRIA, Lyon, France

**Keywords:** BWT, LCP array, SNPs, Reference-free, Assembly-free

## Abstract

**Background:**

Sequencing technologies keep on turning cheaper and faster, thus putting a growing pressure for data structures designed to efficiently store raw data, and possibly perform analysis therein. In this view, there is a growing interest in alignment-free and reference-free variants calling methods that only make use of (suitably indexed) raw reads data.

**Results:**

We develop the *positional clustering* theory that (i) describes how the extended Burrows–Wheeler Transform (eBWT) of a collection of reads tends to cluster together bases that cover the same genome position (ii) predicts the size of such clusters, and (iii) exhibits an elegant and precise LCP array based procedure to locate such clusters in the eBWT. Based on this theory, we designed and implemented an alignment-free and reference-free SNPs calling method, and we devised a consequent SNPs calling pipeline. Experiments on both synthetic and real data show that SNPs can be detected with a simple scan of the eBWT and LCP arrays as, in accordance with our theoretical framework, they are within clusters in the eBWT of the reads. Finally, our tool intrinsically performs a reference-free evaluation of its accuracy by returning the coverage of each SNP.

**Conclusions:**

Based on the results of the experiments on synthetic and real data, we conclude that the positional clustering framework can be effectively used for the problem of identifying SNPs, and it appears to be a promising approach for calling other type of variants directly on raw sequencing data.

**Availability:**

The software ebwt2snp is freely available for academic use at: https://github.com/nicolaprezza/ebwt2snp.

## Background

Sequencing technologies keep on turning cheaper and faster, producing huge amounts of data that put a growing pressure on data structures designed to store raw sequencing information, as well as on efficient analysis algorithms: collections of billion of DNA fragments (reads) need to be efficiently indexed for downstream analysis. The most traditional analysis pipeline after a sequencing experiment, begins with an error-prone and lossy mapping of the reads onto a reference genome. Among the most widespread tools to align reads on a reference genome we can mention BWA [1], Bowtie2 [2], SOAP2 [3]. These methods share the use of the FM-index [4], an indexing machinery based on the Burrows–Wheeler Transform (BWT) [5]. Other approaches [6, 7] combine an index of the reference genome with the BWT of the reads collection in order to boost efficiency and accuracy. In some applications, however, aligning reads on a reference genome presents limitations mainly due to the difficulty of mapping highly repetitive regions, especially in the event of a low-quality reference genome, not to mention the cases in which the reference genome is not even available.

For this reason, indices of reads collections have also been suggested as a lossless dictionary of sequencing data, where sensitive analysis methods can be directly applied without mapping the reads to a reference genome (thus without needing one), nor assembling [8–11]. In [12] the BWT, or more specifically its extension to string collections (named eBWT [13, 14]), is used to index reads from the 1000 Genomes Project [15] in order to support *k*-mer search queries. An eBWT-based compressed index of sets of reads has also been suggested as a basis for both RNA-Seq [16] and metagenomics [17] analyses. There exist also suffix array based data structures devised for indexing reads collections: the *Gk* array [18, 19] and the PgSA [20]. The latter does not have a fixed *k*-mer size. The tool SHREC [21] also uses a suffix-sorting-based index to detect and correct errors in sets of reads. The main observation behind the tool is that sequencing errors disrupt unary paths at deep levels of the reads’ suffix trie. The authors provide a statistical analysis allowing to detect such branching points. Finally, there are several tools [8–11, 22–24] that share the idea of using the de Bruijn graph (dBG) of the reads’ *k*-mers. The advantages of dBG-based indices include allowing therein the characterization of several biologically-interesting features of the data as suitably shaped and sized *bubbles*^1^ (e.g. SNPs, INDELs, alternative splicing events on RNA-Seq data, sequencing errors can all be modeled as bubbles in the dBG of sequencing data [8, 9, 22–24]). The drawback of these dBG representation, as well as those of suffix array based indices [18, 19], is the lossy aspect of getting down to *k*-mers rather than representing the actual whole collection of reads. Also [6, 7] have this drawback as they index *k*-mers. An eBWT-based indexing method for reads collections, instead, has the advantages to be easy to compress and, at the same time, lossless: (e)BWT indexes support querying *k*-mers without the need to build different indexes for different values of *k*.

We introduce the *positional clustering* framework: an eBWT-based index of reads collections where we give statistical characterizations of (i) read suffixes prefixing the same genome’s suffix as *clusters* in the eBWT, and (ii) the onset of these clusters by means of the LCP. This clustering allows to locate and investigate, in a lossless index of reads collections, genome positions possibly equivalent to bubbles in the dBG [8, 22] *independently* from the k-mer length (a major drawback of dBG-based strategies). We thus gain the advantages of dBG-based indices while maintaining those of (e)BWT-based ones. Besides, the eBWT index also contains abundance data (useful to distinguish errors from variants, as well as distinct variant types) and does not need the demanding read-coherency check at post processing as no micro-assembly has been performed. To our knowledge, SHREC [21] and the positional clustering probability framework we introduce in "eBWT positional clustering" subsection , are the only attempts to characterize the statistical behavior of suffix trees of reads sets in presence of errors. We note that, while the two solutions are completely different from the algorithmic and statistical points of view, they are also, in some sense, complementary: SHREC characterizes errors as branching points at deep levels of the suffix trie, whereas our positional framework characterizes clusters of read suffixes prefixing the same genome’s suffix, and identifies mutations (e.g. sequencing errors or SNPs) in the characters *preceding* those suffixes (i.e. eBWT characters). We note that our cluster characterization could be used to detect the suffix trie level from where sequencing errors are detected in SHREC. Similarly, SHREC’s characterization of errors as branching points could be used in our framework to detect further mutations in addition to those in the eBWT clusters.

We apply our theoretical framework to the problem of identifying SNPs. We describe a tool, named ebwt2snp, designed to detect positional clusters and post-process them for assembly-free and reference-free SNPs detection directly on the eBWT of reads collection. Among several reference-free SNPs finding tools in the literature [8, 11, 25, 26], the state-of-the-art is represented by the well documented and maintained KisSNP and DiscoSnp suite [8, 25, 27], where DiscoSnp++ [26] is the latest and best performing tool. In order to validate the accuracy of positional clustering for finding SNPs, we compared DiscoSnp++ sensitivity and precision to those of ebwt2snp by simulating a ground-truth set of SNPs and a read collection. We moreover performed experiments on a real human dataset in order to evaluate the performance of our tool in a more realistic scenario. Results on reads simulated from human chromosomes show that, for example, using coverage 22× our tool is able to find 91% of all SNPs (vs 70% of DiscoSnp++) with an accuracy of 98% (vs 94% of DiscoSnp++). On real data, an approximate ground truth was computed from the raw reads set using a standard aligner-based pipeline. The sensitivity of DiscoSnp++ and ebwt2snp turn out to be similar against this ground-truth (with values ranging from 60 to 85%, depending on the filtering parameters), but, in general, ebwt2snp finds more high-covered SNPs not found by the other two approaches.

A preliminary version of this paper appeared in [28] with limited experiments performed with a prototype tool. This version includes an extension of our strategy to diploid organisms, results on a real dataset, and a new pipeline to generate a .vcf file from our output in the case a reference genome is available.

## Preliminaries

In this section, we define some general terminology we will use throughout this paper. Let $$\Sigma =\{c_1, c_2, \ldots , c_\sigma \}$$ be a finite ordered alphabet with $$c_1< c_2< \cdots < c_\sigma $$, where < denotes the standard lexicographic order. For $$s\in \Sigma ^*$$, we denote its letters by $$s[1], s[2],\ldots ,s[n]$$, where *n* is the *length* of *s*, denoted by |*s*|. We append to $$s\in \Sigma ^*$$ an end-marker symbol $ that satisfies $ $$< c_1$$. Note that, for $$1 \le i \le n$$, $$s[i]\in \Sigma $$ and $$s[n+1]={\$}$$ $$\notin \Sigma $$. A *substring* of *s* is denoted as $$s[i,j] = s[i] \cdots s[j]$$, with *s*[1, *j*] being called a *prefix* and $$s[i,n+1]$$ a *suffix* of *s*.

We denote by $$\mathcal {S} =\{R_1,R_2,\ldots ,R_{m}\}$$ a collection of *m* strings (reads), and by $$\$_i$$ the end-marker appended to $$R_i$$ (for $$1\le i \le m$$), with $$$_i<$$$$$_j$$ if $$i<j$$. Let us denote by *P* the sum of the lengths of all strings in $$\mathcal {S} $$. The *generalized suffix array* GSA of the collection $$\mathcal {S} $$ (see [29–31]) is an array containing *P* pairs of integers (*r*, *j*), corresponding to the lexicographically sorted suffixes $$R_{r}[j, |R_r|+1]$$, where $$1 \le j \le |R_r|+1$$ and $$1 \le r \le m$$. In particular, $${ \textsf {gsa}} (\mathcal {S})[i]=(r,j)$$ (for $$1 \le i \le P$$) if the suffix $$R_{r}[j, |R_r|+1]$$ is the *i*-th smallest suffix of the strings in $$\mathcal {S} $$. Such a notion is a natural extension of the suffix array of a string (see [32]). The Burrows–Wheeler Transform (BWT) [5], a well known text transformation largely used for data compression and self-indexing compressed data structure, has also been extended to a collection $$\mathcal {S} $$ of strings (see [13]). Such an extension, known as *extended Burrows–Wheeler Transform* (eBWT) or multi-string BWT, is a reversible transformation that produces a string that is a permutation of the letters of all strings in $$\mathcal {S} $$: the eBWT of a collection $$\mathcal {S} $$ is denoted by $${ \textsf {ebwt}} (\mathcal {S})$$, and is obtained by concatenating the symbols cyclically preceding each suffix in the list of lexicographically sorted suffixes of all strings in $$\mathcal {S} $$. The eBWT of a collection $$\mathcal {S} $$ can also be defined in terms of the generalized suffix array of $$\mathcal {S} $$ [14]: if $${ \textsf {gsa}} (\mathcal {S})[i]=(j,t)$$ then $${ \textsf {ebwt}} (\mathcal {S})[i] = R_j[t-1]$$; when $$t=1$$, then $${ \textsf {ebwt}} (\mathcal {S})[i]=\$_j$$. For $${ \textsf {gsa}} $$, $${ \textsf {ebwt}} $$, and $${ \textsf {lcp}} $$, the *LF* mapping (resp. *FL*) is a function that associates to each (e)BWT symbol the position preceding (resp. following) it on the text.

The *longest common prefix* (LCP) array of a collection $\mathcal {S}$ of strings (see [30, 31, 33]), denoted by ${ \textsf {lcp}} (\mathcal {S})$, is an array storing the length of the longest common prefixes between two consecutive suffixes of $\mathcal {S}$ in lexicographic order. For each $i=2, \ldots , P$, if ${ \textsf {gsa}} (\mathcal {S})[i-1]=(p_1,p_2)$ and ${ \textsf {gsa}} (\mathcal {S})[i]=(q_1,q_2)$, ${ \textsf {lcp}} (\mathcal {S})[i]$ is the length of the longest common prefix of suffixes starting at positions $p_2$ and $q_2$ of the strings $R_{p_1}$ and $R_{q_1}$, respectively. We set ${ \textsf {lcp}} (\mathcal {S})[1]=0$.

For ${ \textsf {gsa}}$, ${ \textsf {ebwt}}$, and ${ \textsf {lcp}}$, the set $\mathcal {S}$ will be omitted when clear from the context.

## Methods

In this section, we describe our strategy that, given a set of reads sequenced from a genome, allows to find reads clusters with shared context ("eBWT positional clustering" subsection). Moreover, we show how this theoretical framework can be used to design a tool for SNPs detection ("A pipeline for SNPs detection" subsection). Our approach is alignment-free and reference-free, as it does not need to align the reads among each other nor map them on a reference genome: it only makes use of eBWT, LCP and GSA of the reads collection.

### eBWT positional clustering

Let *R* be a read sequenced from a genome *G*[1, *n*]. We say that *R*[*j*] is a *read-copy* of *G*[*i*] iff *R*[*j*] is copied from *G*[*i*] during the sequencing process (and then possibly changed due to sequencing errors). Let us consider the eBWT of a set of reads $\{R_1,\ldots , R_m\}$ of length^2^ *r*, sequenced from a genome *G*. Assuming that *c* is the *coverage* of *G*[*i*], let us denote with $R_{i_1}[j_1], \dots , R_{i_c}[j_c]$ the *c* read-copies of *G*[*i*]. Should not there be any sequencing error, if we consider *k* such that the genome fragment $G[i+1,i+k]$ occurs only once in *G* (that is, nowhere else than right after *G*[*i*]) and if *r* is large enough so that with high probability each $R_{i_t}[j_t]$ is followed by at least *k* nucleotides, then we observe that the *c* read copies of *G*[*i*] would appear contiguously in the eBWT of the reads. We call this phenomenon eBWT* positional clustering*.

We make the following assumptions: (i) the sequencing process is uniform, i.e. the positions from where each read is sequenced are uniform and independent random variables (ii) the probability $\epsilon$ that a base is subject to a sequencing error is a constant (iii) a sequencing error changes a base to a different one uniformly (i.e. with probability 1/3 for each of the three possible variants), and (iv) the number *m* of reads is large (hence, in our theoretical analysis we can assume $m\rightarrow \infty$).

#### **Definition 3.1**

(*eBWT cluster*) The eBWT* cluster of i*, with $1\le i \le n$ being a position on *G*, is the substring ${ \textsf {ebwt}} [a,b]$ such that ${ \textsf {gsa}} [a,b]$ is the range of read suffixes prefixed by $G[i+1,i+k]$, where $k<r$ is the smallest value for which $G[i+1,i+k]$ appears only once in *G*. If no such value of *k* exists, we take $k=r-1$ and say that the cluster is *ambiguous*.

If no value $k<r$ guarantees that $G[i+1,i+k]$ appears only once in *G*, then the eBWT cluster of *i* does not contain only read-copies of *G*[*i*] but also those of other $t-1$ characters $G[i_2], \dots , G[i_t]$. We call *t* the *multiplicity* of the eBWT cluster. Note that $t=1$ for non-ambiguous clusters.

Due to sequencing errors, and to the presence of repetitions with mutations in real genomes, a *clean* eBWT positional clustering is not realistic. However, we show that, even in the event of sequencing errors, in the eBWT of a collection of reads sequenced from a genome *G*, the read-copies of *G*[*i*] still *tend to be clustered* together according to a suitable Poisson distribution.

#### **Theorem 3.2**

(eBWT positional clustering) *Let* $R_{i_1}[j_1], \dots , R_{i_c}[j_c]$ *be the* *c* *read-copies of* *G*[*i*]. *An expected number* $X\le c$ *of these read copies will appear in the* eBWT *cluster* ${ \textsf {ebwt}} [a,b]$ *of* *i*, *where* $X\sim Poi(\lambda )$ *is a Poisson random variable with mean*

$$\begin{aligned} \lambda = m\cdot \frac{r-k}{n}\left( 1-\epsilon \right) ^{k} \end{aligned}$$

*and where* *k* *is defined as in Definition* 3.1.

#### *Proof*

The probability that a read covers *G*[*i*] is *r*/*n*. However, we are interested only in those reads such that, if *R*[*j*] is a read-copy of *G*[*i*], then the suffix $R[j+1,r+1]$ contains at least *k* nucleotides, i.e. $j \le r-k$. In this way, the suffix $R[j+1,r+1]$ will appear in the GSA range ${ \textsf {gsa}} [a,b]$ of suffixes prefixed by $G[i+1, i+k]$ or, equivalently, *R*[*j*] will appear in ${ \textsf {ebwt}} [a,b]$. The probability that a random read from the set is uniformly sampled from such a position is $(r-k)/n$. If the read contains a sequencing error inside $R[j+1,j+k]$, however, the suffix $R[j+1,r+1]$ will not appear in the GSA range ${ \textsf {gsa}} [a,b]$. The probability that this event does not happen is $(1-\epsilon )^k$. Since we assume that these events are independent, the probability of their intersection is therefore

$$\begin{aligned} Pr(R[j] \in { \textsf {ebwt}} [a,b]) = \frac{r-k}{n}\left( 1-\epsilon \right) ^{k} \end{aligned}$$

This is a Bernoullian event, and the number *X* of read-copies of *G*[*i*] falling in ${ \textsf {ebwt}} [a,b]$ is the sum of *m* independent events of this kind. Then, *X* follows a Poisson distribution with mean $\lambda = m\cdot \frac{r-k}{n}\left( 1-\epsilon \right) ^{k}$. $\square$

Theorem 3.2 states that, if there exists a value $k<r$ such that $G[i+1, i+k]$ appears only once in *G* (i.e. if the cluster of *i* is not ambiguous), then *X* of the $b-a+1$ letters in ${ \textsf {ebwt}} [a,b]$ are read-copies of *G*[*i*]. The remaining $(b-a+1)-X$ letters are noise introduced by suffixes that mistakenly end up inside ${ \textsf {gsa}} [a,b]$ due to sequencing errors. It is not hard to show that this noise is extremely small under the assumption that *G* is a uniform text; we are aware that this assumption—as well as those required by Theorem 3.2—is not completely realistic, but we will experimentally show in "Experimental evaluation" section that also on real datasets our approach produces accurate results (as predicted by our simplified theoretical framework). See "SNP calling (ebwt2snp)" subsection for our complete strategy in a real scenario.

Note that the expected coverage of position *G*[*i*] is also a Poisson random variable, with mean $\lambda ' = \frac{mr}{n}$ equal to the average coverage. On expectation, the size of non-ambiguous eBWT clusters is thus $\lambda /\lambda ' = \frac{(r-k)(1-\epsilon )^k}{r} <1$ times the average coverage. E.g., with $k=14$, $\epsilon =0.0033$ (see [34, Table 1, HiSeq, R2]), and $r=100$ the expected cluster size is $100\cdot \lambda /\lambda ' \approx 80\%$ the average coverage.

Finally, it is not hard to prove, following the proof of Theorem 3.2, that in the general case with multiplicity $t\ge 1$ the expected cluster size follows a Poisson distribution with mean $t\cdot \lambda$ (because the read-copies of *t* positions are clustered together).

Note that in this section we use the reference genome for our theoretical analysis only. In practice, the reference genome could be unknown, and our tool (described in the next sections) will not need it.

So far, we have demonstrated the eBWT positional clustering property but we don’t have a way to efficiently locate the eBWT clusters. A naive strategy could be to fix a value of *k* and define clusters to be ranges of *k*-mers in the GSA. This solution, however, fails to separate read suffixes differing after *k* positions (this is, indeed, a drawback of all *k*-mer-based strategies). The aim of Theorem 3.3 is precisely to fill this gap, allowing us to move from theory to practice. Intuitively, we show that clusters lie between local minima in the LCP array. This strategy automatically detects, in a data-driven way, the value *k* satisfying Definition 3.1 (crucially, *k* is not the same for all clusters).

Our result holds if two conditions on the cluster ${ \textsf {ebwt}} [a,b]$ of a position under investigation are satisfied:

1. The cluster does not have noise, i.e. $X = b-a+1$, and
2. Let $(p_1,j_1), (p_2,j_2) \in { \textsf {gsa}} [a,b]$. For any *x* such that $k\le x<r$, if both $R_{p_1}[j_1,r]$ and $R_{p_2}[j_2,r]$ contain their leftmost sequencing errors in $R_{p_1}[j_1+x]$ and $R_{p_2}[j_2+x]$, then $R_{p_1}[j_1+x] \ne R_{p_2}[j_2+x]$.

Intuitively, Condition (2) states that two sequencing errors on two read-copies of the same genome letter do not produce the same nucleotide (intuitively, this is unlikely to happen with low sequencing error rates and not too high coverages). In [28, Prop. 4] we proved that with probability high enough Condition (2) is satisfied in practice.

#### **Theorem 3.3**

*Let* ${ \textsf {ebwt}} [a,b]$ *be the* eBWT *cluster of a position* *i* *meeting Conditions (1) and (2). Then, there exists a value* $a < p \le b$ *such that* ${ \textsf {lcp}} [a+1,p]$ *is a non-decreasing sequence and* ${ \textsf {lcp}} [p+1,b]$ *is a non-increasing sequence*.

#### *Proof*

Let us denote by $p_M$ the largest index in (*a*, *b*] such that ${ \textsf {lcp}} [p_M]=M$, where *M* is the maximum value of LCP in (*a*, *b*] (if *M* occurs multiple times, take the rightmost occurrence). We claim the theorem holds for $p=p_M$. Let us denote by *q* and *j*, $1\le q \le m$, $1\le j \le r$, the positive integers such that ${ \textsf {gsa}} [p_M]=(q,j)$. This means, by using Condition (2), that the read $R_q$ contains the longest prefix $R_q[j,j+M-1]$ without sequencing errors, $j+M\le r+1$. Consider any other suffix $R_u[j_u,r+1]$ in the range and let $R_u[j_u+x]$ be the leftmost mismatch letter in $R_u[j_u,r+1]$, with $x\ge k$. Note that if $R_u[j_u,r+1]$ does not contain sequencing errors, then $j_u+x-1=r$, otherwise $R_u[j_u+x]$ has been mutated with an error. We suppose that the mutation at position $j_u+x$ generated a letter lexicographically smaller than that of the genome (the other case is symmetric). By Condition (2), $x\le M+1$ and no other suffix $R_{v}[j_v,r+1]$ in the range satisfies $R_{v}[j_{v}+x] = R_u[j_u+x]$. Then, $R_u[j_u,r+1]$ falls right after a suffix $R_{u'}[j_{u'},r+1]$ such that either $R_{u'}[j_{u'},r]$ properly prefixes $R_u[j_u,r+1]$ or the leftmost mismatch occurs at position $x'\le x$. Similarly, $R_u[j_u,r+1]$ falls right before a suffix $R_{u''}[j_{u''},r+1]$ such that $R_{u}[j_{u}+x] < R_{u''}[j_{u''}+x]$ and whose leftmost mismatch position $x''$ satisfies $x\le x''\le M+1$. This shows that, before suffix $R_q[j,r+1]$, other suffixes are ordered by increasing position of their leftmost mismatch letter (since $x'\le x \le x''$) and, then, by the lexicographic order among mismatch letters, which in particular implies that before suffix $R_q[j,r+1]$ the lcp values are non-decreasing. Symmetrically, with a similar reasoning one can easily prove that after suffix $R_q[j,r+1]$ the lcp values are non-increasing. $\square$

According to Theorem 3.3, clusters are delimited by local minima in the LCP array of the reads set. This gives us a strategy for finding clusters that is *independent* from *k*. Importantly, the proof of Theorem 3.3 also gives us the suffix in the range (the *p*-th suffix) whose longest prefix without sequencing errors is maximized. As we will show in the next section, this will be useful to efficiently compute a consensus of the reads in the cluster.

Observe that by applying Theorem 3.3 we also find ambiguous clusters. However, the expected length of these clusters is a multiple of $\lambda$, so they can be reliably discarded with a significance test based on the Poisson distribution of Theorem 3.2.

### A pipeline for SNPs detection

When the reads dataset contains variations (e.g. two allele of the same individual, or two or more distinct individuals, or different isoforms of the same gene in RNA-Seq data, or different reads covering the same genome fragment in a sequencing process, etc.), the eBWT positional clustering described in "eBWT positional clustering" subsection can be used to detect, directly from the raw reads (hence, without assembly and without the need of a reference genome), positions *G*[*i*] exhibiting possibly different values, but followed by the same context: they will be in a cluster delimited by LCP minima and containing possibly different letters (corresponding to the read copies of the variants of *G*[*i*] in the reads set). We now describe how to use this theoretical framework to discover SNPs just scanning eBWT, LCP and GSA of the sets of reads, without aligning them nor mapping them onto a reference genome.

Since (averagely) half of the reads comes from the forward (F) strand, and half from the reverse-complement (RC) strand, we denote with the term *right* (resp. *left*) *breakpoint* those variants found in a cluster formed by reads coming from the F (resp. RC) strand, and therefore sharing the right (resp. left) context adjacent to the variant. A *non-isolated SNP* [25] is a variant at position *i* such that the closest variant is within *k* bases from *i*, for some fixed *k* (we use $k=31$ in our validation procedure, see below). The SNP is *isolated* otherwise. Note that, while isolated SNPs are found twice with our method (one as a right breakpoint and one as a left breakpoint), this is not true for non-isolated SNPs: variants at the sides of a group of non-isolated SNPs are found as either left or right breakpoint, while SNPs *inside* the group will be found with positional clustering plus a partial local assembly of the reads in the cluster. In the next two subsections we give all the details of our strategy.

**Fig. 1:**
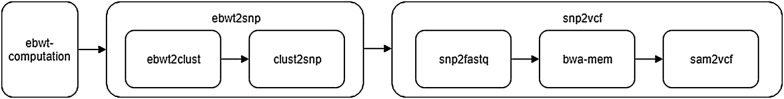
Our complete pipeline, including pre-processing and post-processing phases

Our main suite performing the SNP-calling step is called ebwt2snp, and is described in more detail in "SNP calling (ebwt2snp)" subsection. ebwt2snp requires a pre-processing phase to be executed beforehand (described in "Pre-processing (eBWT computation)" subsection), where we compute the required data structures. If a reference genome is available, after the execution of ebwt2snp one can further run a post-processing phase called snp2vcf (described in "Post-processing (snp2vcf)" subsection) in order to obtain a .vcf file containing the identified SNPs. Figure 1 depicts the whole pipeline.

#### Pre-processing (eBWT computation)

Since we do not aim at finding matching pairs of clusters on the forward and reverse strands, we augment the input adding the reverse-complement of the reads: for a reads set $\mathcal {S}$, we add $\mathcal {S} ^{RC}$ as well. Hence, given two reads sets $\mathcal {S}$ and $\mathcal {T}$, in the pre-processing phase we compute ${ \textsf {ebwt}} (\mathcal {R})$, ${ \textsf {lcp}} (\mathcal {R})$, and ${ \textsf {gsa}} (\mathcal {R})$, for $\mathcal {R} = \{ \mathcal {S} \cup \mathcal {S} ^{RC} \cup \mathcal {T} \cup \mathcal {T} ^{RC}\}$. This task can be achieved using, for example, BCR^3^ [30], eGSA^4^ [31] or gsacak^5^ [35]. We also compute ${ \textsf {gsa}} (\mathcal {R})$ because we will need it (see "SNP calling (ebwt2snp)" subsection) to extract left and right contexts of the SNP. Though this could be achieved by performing (in external memory) multiple steps of LF- and FL-mappings on the eBWT, this would significantly slow down our tool. Note that our approach can also be generalized to more than two reads collections.

#### SNP calling (ebwt2snp)

Our SNPs calling approach takes as input ${ \textsf {ebwt}} (\mathcal {R})$, ${ \textsf {lcp}} (\mathcal {R})$, and ${ \textsf {gsa}} (\mathcal {R})$ and outputs SNPs in KisSNP2 format [27]: a fasta file containing a pair of sequences per SNP (one per sample, containing the SNP and its context). The SNP calling, implemented in the ebwt2snp suite, is composed by the following modules (to be executed sequentially): ebwt2clust and clust2snp.

ebwt2clust: partitions ${ \textsf {ebwt}} (\mathcal {R})$ in clusters corresponding to the same genome position as follows. A scan of ${ \textsf {ebwt}} (\mathcal {R})$ and ${ \textsf {lcp}} (\mathcal {R})$ finds clusters using Theorem 3.3, and stores them as a sequence of ranges of the eBWT. While computing the clusters, we also apply a threshold of minimum LCP (by default, 16), cutting clusters tails with LCP values below the threshold; this filtering drastically reduces the number of stored clusters (and hence memory usage and running time), avoiding to output many short clusters corresponding to noise. The outputs is a .clusters file.

clust2snp: it takes as input the clusters file produced by ebwt2clust, ${ \textsf {ebwt}} (\mathcal {R})$, ${ \textsf {lcp}} (\mathcal {R})$, ${ \textsf {gsa}} (\mathcal {R})$, and $\mathcal {R}$, processing clusters from first to last as follows:

1. We compute empirically the cluster size distribution. Experimentally, we observed that this distribution has exactly the mean predicted by Theorem 3.2. However, due to the fact that on real data the coverage is not uniform (as required by the assumptions of Theorem 3.2), we observed a higher variance with respect to the Poisson distribution of Theorem 3.2. For this reason, in practice we refer to the empirical observed distribution of cluster sizes, rather than the theoretical one.
2. We test the cluster’s length using the distribution computed in step 1; if the cluster’s length falls in one of the two tails at the sides of the distribution (by default, the two tails summing up to 5% of the distribution), then the cluster is discarded; moreover, due to *k*-mers that are not present in the genome but appear in the reads because of sequencing errors (that introduce noise around cluster length equal to 1), we also fix a minimum value of length for the clusters (by default, four letters per sample).
3. In the remaining clusters, we find the most frequent nucleotides $b_1$ and $b_2$ of samples 1 and 2, respectively, and check whether $b_1 \ne b_2$; if so, then we have a candidate SNP: for each sample, we use the GSA to retrieve the coordinate of the read containing the longest right-context without errors; moreover, we retrieve, and temporarily store in a buffer, the coordinates of the remaining reads in the cluster associated with a long enough LCP value (by default, at least $k=30$ bases). For efficiency reasons, the user can also specify an upper bound to the number of reads to be extracted. In case of diploid samples and heterozygous sites, up to two nucleotides $b^1_i, b^2_i$ per individual ($i=1,2$ being the individual’s index) are selected (i.e. the two most frequent), and we repeat the above procedure for any pair of nucleotides $b^{j'}_1 \ne b^{j''}_2$ exhibiting a difference among the two individuals.
4. After processing all events, we scan the fasta file storing $\mathcal {R}$ to retrieve the reads of interest (those whose coordinates are in the buffer); for each cluster, we compute a consensus of the read fragments preceding the SNP, for each of the two samples. This allows us to compute a left-context for each SNP (by default, of length $k+1=31$), and it also represents a further validation step: if the assembly cannot be built because a consensus cannot be found, then the cluster is discarded. The number *C* of reads in accordance with the computed consensus (i.e. within small Hamming distance—by default 2—from the consensus) is also stored to output. This value can be used to filter the output at post-processing time (i.e. to require that each SNP is supported by at least a certain number of reads). Note that these left-contexts preceding SNPs (which are actually right-contexts if the cluster is formed by reads from the RC strand) allow us to capture non-isolated SNPs. Each SNP is returned as a pair of DNA fragments (one per sample) of length $2k+1$ (where, by default, $k=30$), with the SNP in the middle position.

The output of clust2snp is a .snp file (this is actually a fasta file containing pairs of reads testifying the variations). We remark that, given the way our procedure is defined, the fragments of length $2k+1$ that we output are always substrings (at small Hamming distance—by default, 2) of at least *C* reads (*C* being the above-mentioned number of reads in accordance with the computed consensus). This means that our method cannot output chimeric fragments: all SNPs we output are effectively supported by at least a certain number of reads. This number is stored to output and can be used to filter the result at post-processing time.

#### Post-processing (snp2vcf)

Finally, for the cases where a reference genome is available, we have designed a second pipeline snp2vcf that processes the results of ebwt2snp to produce a .vcf file^6^. Since the input of ebwt2snp is just a reads set, the tool cannot directly obtain the SNPs positions (in the genome) required for building the .vcf file. For this, we need a reference genome and an alignment tool.

snp2fastq: Converts the .snp file produced by clust2snp into a .fastq file (with dummy base qualities) ready to be aligned.
bwa-mem^7^: Is a well-known tool that maps low-divergent sequences against a large reference genome [1, 36]. The output is a .sam file.
sam2vcf: Converts the .sam file produced in the previous step into a .vcf file containing the variants.

#### Complexity

In the clustering step, we process the eBWT and LCP and on-the-fly output clusters to disk. The SNP-calling step performs one scan of the eBWT, GSA, and clusters file to detect interesting clusters, plus one additional scan of the reads set to retrieve contexts surrounding SNPs. Both these phases take linear time in the size of the input and do not use disk space in addition to the input and output. Due to the fact that we store in a buffer the coordinates of reads inside interesting clusters, this step uses an amount of RAM proportional to the number of SNPs times the average cluster size $\lambda$ times the read length *r* (e.g. a few hundred MB in our case study of "Experimental evaluation" section). Notice that our method is very easy to parallelize, as the analysis of each cluster is independent from the others.

## Experimental evaluation

In this section we test the performance of our method using simulated ("Experiments on real data" subsection) and real ("Experiments on synthetic data" subsection) datasets. In the first case, the starting point is the ground truth, that is a real .vcf file, while the synthetic data is consequently generated, starting from a real sequence, using such file and a sequencing simulator. In the second case, the starting point is real raw reads data for which the real ground truth is not available, and hence, in order to validate our results, we have generated a synthetic one by means of a standard pipeline. Note that, since the use of a synthetic ground truth can generate errors, our approach is also able to provide a further estimate of the accuracy of the identified SNPs, on the basis of the number of reads needed to identify them, as detailed in the following.

We compare ebwt2snp with DiscoSnp++, that is an improvement of the DiscoSnp algorithm: while DiscoSnp only detects (both heterozygous and homozygous) *isolated* SNPs from any number of read datasets without a reference genome, DiscoSnp++ detects and ranks all kinds of SNPs as well as small indels. As shown in [26], DiscoSnp++ performs better than state-of-the-art methods in terms of both computational resources and quality of the results.

DiscoSnp++ is a pipeline of several independent tools. As a preprocessing step, the dBG of the input datasets is built, and presumed erroneous *k*-mers are removed. Then, DiscoSnp++ detects bubbles generated by the presence of SNPs (isolated or not) and indels, and it outputs a fasta file containing the variant sequences (KisSNP2 module). A final step (kissreads2) maps back the reads from all input reads sets on the variant sequences, mainly in order to determine the read coverage per allele and per reads set of each variant. This module also computes a rank per variant, indicating whether it exhibits discriminant allele frequencies in the datasets. The last module generates a .vcf of the predicted variants. If no reference genome is provided, this step is simply a change of format from fasta to .vcf (VCFcreator module).

Our framework has been implemented in C++ and is available at https://github.com/nicolaprezza/ebwt2snp. All tests were done on a DELL PowerEdge R630 machine, used in non exclusive mode. Our platform is a 24-core machine with Intel(R) Xeon(R) CPU E5-2620 v3 at 2.40 GHz, with 128 GB of shared memory. The system is Ubuntu 14.04.2 LTS. Notice that a like-for-like comparison of the time consumption between our implementation and DiscoSnp++ is not possible, since DiscoSnp++ is multi-thread and our tool is currently designed to use one core only. For instance, on the real dataset, DiscoSnp++ (in the case where $b=1$) needs about 17-18 hours for computing the SNPs when only one core is used (where the percentage of CPU usage got equal to 99%) rather than 2 h with multi-threading enabled (where the percentage of CPU usage got equal to 1, 733%). DiscoSnp++ needs, for the construction of the de Bruijn graph in the preprocessing phase, about 32 min with multi-threading enabled (where the percentage of CPU usage got equal to 274%) rather than about 1 h and 19 min when only one core is used (where the percentage of CPU got equal to 99%).

We experimentally observed that the pre-processing step (see Table 1) is more computationally expensive than the actual SNP calling step. The problem of computing the eBWT is being intensively studied, and improving its efficiency is out of the aim of this paper. However, a recent work [12] suggests that directly storing raw read data with a compressed eBWT leads to considerable space savings, and could therefore become the standard in the future. Our strategy can easily be adapted to directly take as input these compressed formats (which, as opposed to data structures such as the de Bruijn graph, are lossless file representations and therefore would replace the original read set). Building the dBG requires a few minutes (using multicore) and, in order to keep the RAM usage low, no other information other than *k*-mer presence is stored in the dBG used by DiscoSnp++. On the other hand, the construction of the eBWT, LCP and GSA arrays can take several hours (using a single core). As a consequence, overall DiscoSnp++ is faster than our pipeline when also including pre-processing. Further extensions of this work will include removing the need for the GSA/LCP arrays, which at the moment represent a bottleneck in the construction phase, and taking as input a compressed eBWT.

### Experiments on synthetic data

We propose a first experiment simulating two human chromosomes haploid reads sets obtained mutating (with *real* .vcf files) *real* reference chromosomes^8^. The final goal of the experiments is to reconstruct the variants contained in the original (ground truth) .vcf files. We generated the mutated chromosomes using the 1000 genome project (phase 3) .vcf files^9^ related to chromosomes 16 and 22, suitably filtered to keep only SNPs of individuals HG00100 (ch.16) and HG00096 (ch.22). From these files, we simulated Illumina sequencing with SimSeq [37], both for reference and mutated chromosomes: individual HG00096 (ch.22) at a 29× getting 15,000,000 reads of length 100-bp, and individual HG00100 (ch.16) a 22× getting 20,000,000 reads of length 100-bp. To simulate the reads, we used the HiSeq error profile^10^ publicly available in the SimSeq’s repository. Note that our experiments, including the synthetic data generation, are easily reproducible given the links of the datasets, simulator, and error profile we have provided.

**Table 1:** Pre-processing comparative results of ebwt2snp (i.e. building the eBWT using either eGSA or BCR) and DiscoSnp++ (i.e. building the de Bruijn graph)

Dataset	Coverage per sample	#reads	Preprocessing	Wall clock^a^ (h:mm:ss)	RAM (MB)
HG00096 (ch. 22)	29×	15,000,000	gsacak	0:53:34	100,607
			eGSA	1:41:37	30,720
			BCR	4:18:00	1,970
			DiscoSnp++	0:01:09	5,170
HG00100 (ch. 16)	22×	20,000,000	gsacak	1:13:04	112,641
			eGSA	3:39:04	30,720
			BCR	6:10:28	3,262
			DiscoSnp++	0:02:01	6,111
HG00419+NA19017 (ch. 1)	43×–47×	93,657,983	BCR	105:28:30	73,977
			DiscoSnp++	0:32:37	621

Wall clock is the elapsed time from start to completion of the instance, while RAM is the peak Resident Set Size (RSS). Both values were taken with /usr/bin/time command. Note that for the last collection we have used a variant of BCR that keeps the ${ \textsf {ebwt}} (\mathcal {S})$ in internal memory. eGSA and gsacak have not been tested on the last dataset since they required too much disk space and RAM, respectively

^a^We recall that DiscoSnp++ makes use of multiple cores while ebwt2snp is currently designed to use one core only, thus explaining the difference in speed

#### Validation

Here we describe the validation tool snp_vs_vcf we designed to measure the sensitivity and precision of any tool returning SNPs in KisSNP2 format. Note that we output SNPs as pairs of reads containing the actual SNPs plus their contexts (one sequence per sample). This can be formalized as follows: the output is a series of pairs of triples (we call them *calls*) $(L',s',R'),\ (L'',s'',R'')$ where $L'$, $R'$, $L''$, $R''$ are the left/right contexts of the SNP in the two samples, and letters $s'$, $s''$ are the actual variant. Given a .vcf file containing the ground truth, the most precise way to validate this kind of output is to check that the triples actually match contexts surrounding true SNPs on the reference genome (used here just for accuracy validation purposes). That is, for each pair in the output calls:

1. If there is a SNP $s'\rightarrow s''$ in the .vcf of the first sample with contexts $L', R'$ (or their RC), then $(L',s',R'),\ (L'',s'',R'')$ is a true positive (TP).
2. Any pair $(L',s',R'),\ (L'',s'',R'')$ that does not match any SNP in the ground truth (as described above) is a false positive (FP).
3. Any SNP in the ground truth that does not match any call is a false negative (FN).

We implemented the above validation strategy with a (quite standard) reduction of the problem to the 2D range reporting problem: we insert in a two-dimensional grid two points per SNP (from the .vcf) using as coordinates the ranks of its right and (reversed) left contexts among the sorted right and (reversed) left contexts of all SNPs (contexts from the first sample) on the F and RC strands. Given a pair $(L',s',R'),\ (L'',s'',R'')$, we find the two-dimensional range corresponding to all SNPs in the ground truth whose right and (reversed) left contexts are prefixed by $R'$ and (the reversed) $L'$, respectively. If there is at least one point in the range matching the variation $s'\rightarrow s''$, then the call is a TP (case 1 above; note that, in order to be a TP, a SNP can be found either on the F or on the RC strand, or both); otherwise, it is a FP (case 2 above). Since other tools such as DiscoSnp++ do not preserve the order of samples in the output, we actually check also the variant $s''\rightarrow s'$ and also search the range corresponding to $L''$ and $R''$. Finally, pairs of points (same SNP on the F/RC strands) that have not been found by any call are marked as FN (case 3 above). We repeat the procedure for any other SNP found between the two strings $L's'R'$ and $L''s''R''$, in order to find non-isolated SNPs.

#### Results

We run DiscoSnp++ with default parameters (hence *k*-mers size set to 31) except for $P=3$ (it searches up to *P* SNPs per bubble) and parameter *b*, for which we ran all the three versions ($b=0$ forbids variants for which any of the two paths is branching; $b=2$ imposes no limitation on branching; $b=1$ is inbetween).

ebwt2snp takes as input few main parameters, among which the most important are the lengths of right and left SNPs contexts in the output (−L and −R), and (−v) the maximum number of non-isolated SNPs to seek in the left contexts (same as parameter *P* of DiscoSnp++). In order to make a fair comparison between DiscoSnp++ and ebwt2snp, with ebwt2snp we decided to output (exactly as for DiscoSnp++) 30 nucleotides following the SNP (-R 30), 31 nucleotides preceding and including the SNP (−L 31) (i.e. the output reads are of length 61, with the SNP in the middle position), and −v 3 (as we used $P=3$ with DiscoSnp++). We validated our calls after filtering the output so that only SNPs supported by at least $cov=4$ and 6 reads were kept.

In Table 2, we show the number of TP, FP and FN as well as sensitivity (SEN), precision (PREC), and the number of non-isolated SNPs found by the tools. The outcome is that ebwt2snp is always more precise and sensitive than DiscoSnp++. Moreover, while in our case precision is stable and always quite high (always between 94 and 99%), for DiscoSnp++ precision is much lower in general, and even drops with $b=2$, especially with lower coverage, when inversely sensitivity grows. Sensitivity of DiscoSnp++ gets close to that of ebwt2snp only in case $b=2$, when its precision drops and memory and time get worse than ours.

**Table 2:** Post-processing comparative results of ebwt2snp (i.e. building clusters from the eBWT and performing SNP calling) and DiscoSnp++ (i.e. running KisSNP2 and kissreads2 using the pre-computed de Bruijn graph)

Tool	Param.	Wall clock	RAM (MB)	TP	FP	FN	SEN (%)	PREC (%)	Non-isol. SNP
Individual HG00096 vs reference (chromosome 22, 50818468bp), coverage 29× per sample									
DiscoSnp++	b = 0	5:07	101	32,773	3719	13,274	71.17	89.81	4707/8658
	b = 1	16:39	124	37,155	10,599	8892	80.69	77.80	5770/8658
	b = 2	20:42	551	40,177	58,227	5870	87.25	40.83	6325/8658
ebwt2snp	$\hbox {cov}=4$	35:56	314	42,309	1487	3738	91.88	96.60	7233/8658
	$\hbox {cov}=6$	22:19	300	40,741	357	5306	88.47	99.13	6884/8658
Individual HG00100 vs reference (chromosome 16, 90338345bp), coverage 22× per sample									
DiscoSnp++	b=0	6:20	200	48,119	10,226	18,001	72.78	82.47	6625/11,055
	b=1	31:57	208	53,456	24,696	12,664	80.85	68.40	7637/11,055
	b=2	51:45	1256	57,767	124,429	8353	87.37	31.71	8307/11,055
ebwt2snp	$\hbox {cov}=4$	33:24	418	59,668	898	6452	90.24	98.51	9287/11,055
	$\hbox {cov}=6$	44:53	337	53,749	190	12,371	81.29	99.64	8169/11,055

Wall clock (mm:ss) is the elapsed time from start to completion of the instance, while RAM is the peak Resident Set Size (RSS). Both values were taken with /usr/bin/time command. We recall that DiscoSnp++ makes use of multiple cores while ebwt2snp is currently designed to use one core only, thus explaining the difference in speed

Note that precision and sensitivity of DiscoSnp++ are consistent with those reported in [26]. In their paper (Table 2), the authors report a sensitivity of $79.31\%$ and a precision of $72.11\%$ for DiscoSnp++ evaluated on a Human chromosome with simulated reads (i.e. using an experimental setting similar to ours). In our experiments, using parameter $b=1$, DiscoSnp++ ’s sensitivity and precision are, on average between the two datasets, $80.77\%$ and $73.1\%$, respectively. Therefore, such results almost perfectly match those obtained by the authors of [26]. The same Table 2 of [26] shows that DiscoSnp++ can considerably increase precision at the expense of sensitivity by filtering low-ranking calls. By requiring $rank>0.2$, the authors show that their tool achieves a sensitivity of $65.17\%$ and a precision of $98.73\%$. While we have not performed this kind of filtering in our experiments, we note that also in this case ebwt2snp’s sensitivity would be higher than that of DiscoSnp++. Precision of the two tools, on the other hand, would be comparable.

Finally, we note that also DiscoSnp++ has been evaluated by the authors of [26] using the SimSeq simulator (in addition to other simulators which, however, yield similar results). We remark that SimSeq simulates position-dependent sequencing errors, while our theoretical assumptions are more strict and require position-independent errors. Similarly, we assume a uniform random genome, while in our experiments we used real human chromosomes. Since in both cases our theoretical assumptions are more stringent than those holding on the datasets, the high accuracy we obtain is a strong evidence that our theoretical analysis is robust to changes towards less-restrictive assumptions.

### Experiments on real data

In order to evaluate the performance of our pipeline on real data, we reconstructed the SNPs between the chromosome 1 of the two 1000 genomes project’s individuals HG00419 and NA19017 using as starting point the high-coverage reads sets available at ftp://ftp.1000genomes.ebi.ac.uk/vol1/ftp/phase3/data/. The two datasets consist of 44,702,373 and 48,955,610 single-end reads, respectively, of maximum length 250 bases. This corresponds to a coverage of 43× and 47× for the two individuals, respectively. The input dataset of our pipeline, which includes the union of these reads and their reverse-complements, summing up to 43 Gb.

Since, in this case, the real ground truth SNP set is not known, we compare the outputs of our tool and DiscoSnp++ against those of a standard SNP-calling pipeline based on the aligner bwa-mem and the post-processing tools samtools, bcftools, and vcftools. We thus developed a validation pipeline that does not rely on a known ground-truth .vcf (which in the real case does not exist). To generate the synthetic ground-truth .vcf, we use a standard (aligner and SNP-caller) pipeline described below.

#### Validation

Our validation pipeline proceeds as follows.

1. We align the reads of the first individual on the human reference’s chromosome 1 (using bwa-mem).
2. From the above alignment file, we compute a .vcf file describing the variations of the first individual with respect to the human reference’s chromosome 1 (using samtools and bcftools).
3. We apply the .vcf to the reference, generating the first individual’s chromosome sequence (using vcftools).
4. We align the reads of the second individual on the first individual sequence obtained at the previous step.
5. From the above alignment, we obtain the “ground-truth” .vcf file containing the variations of the first individual with respect to the second one. Again, for this step we used a pipeline based on samtools and bcftools.
6. We evaluate sensitivity and precision of the file in KisSNP2 format (generated by ebwt2snp or DiscoSnp++) against the ground truth .vcf generated at the previous step. This final validation is carried out using our own module snp_vs_vcf.

The exact commands used to carry out all validation steps can be found in the script pipeline.sh available in our software repository. Note that the accuracy of the aligner/SNP-caller pipeline greatly affects the computed ground truth, which is actually a synthetic ground truth, and (unlike in the simulated datasets) will necessarily contain errors with respect to the real (unknown) ground truth. Note also that ebwt2snp outputs the coverage of each SNP (i.e. how many reads were used to call the SNP). This information can also be used to estimate the output’s precision (i.e. the higher the coverage is, the more likely it is that the SNP is a true positive).

#### Results

**Table 3:** Sensitivity and precision of the ebwt2snp pipeline

Cov	SEN (%)	PREC (%)	TP	FP	FN	Non-isol	Non isol (%)
3	84.34	45.19	317,490	385,060	58,938	45,363	65.34
4	83.18	50.67	313,131	304,811	63,297	44,491	64.08
5	80.53	60.36	303,130	199,042	73,298	42,394	61.06
6	77.94	66.62	293,385	146,972	83,043	40,403	58.20
7	75.22	70.93	283,145	116,042	93,283	38,405	55.32
8	72.32	73.99	272,223	95,675	104,205	36,427	52.47
9	69.18	76.33	260,405	80,746	116,023	34,391	49.54
10	65.80	78.16	247,685	69,203	128,743	32,281	46.50
11	59.83	79.82	225,232	56,929	151,196	28,846	41.55
12	55.45	81.21	208,725	48,284	167,703	26,360	37.97

Values are computed using as ground truth the SNPs predicted by a classic aligner-based pipeline

**Table 4:** Sensitivity and precision of the DiscoSnp++ pipeline

b	Wall clock	RAM (MB)	SEN (%)	PREC (%)	TP	FP	FN	Non-isol	Non isol (%)
0	00:42:46	608	62.62	87.21	235,749	34,547	140,679	18,561	26.73
1	02:13:23	866	71.94	74.57	270,811	92,310	105,617	31,640	45.57
2	11:09:09	13,830	77.31	45.41	291,022	349,754	85,406	34,840	50.18

Values are computed using as ground truth the SNPs predicted by a classic aligner-based pipeline

ebwt2clust terminated in 55 min and used 3 MB of RAM, while clust2snp terminated in 2 h and 43 min and used 12 GB of RAM. Filtering the output by minimum coverage (to obtain the different rows of Table 3) required just a few seconds. The whole ebwt2snp pipeline, pre-processing excluded, required therefore about 3 hours and used 12 GB of RAM.

Table 3 (resp. Table 4) shows the comparison between ebwt2clust (resp. DiscoSnp++) and the SNPs predicted by an aligner-based pipeline.

The results of ebwt2clust are shown with a filtering by minimum coverage ranging from 3 to 12, while DiscoSnp++ performances are shown with parameter *b* ranging from 0 to 2.

With parameter $b=0$, DiscoSnp++ exhibits a sensitivity of $62.62\%$, close to ebwt2clust ’s sensitivity around $cov=11$ (i.e. they output approximately the same number of true positives). With these parameters, DiscoSnp++ outputs less false positives (and has thus higher precision) than ebwt2clust. This is related the fact that ebwt2clust outputs more SNPs (i.e. TP+FP) than DiscoSnp++, and a higher fraction of these SNPs do not find a match in the ground truth and are thus classified as false positive. We stress out that, in this particular case, each SNP output by ebwt2clust is covered by at least 22 reads (at least 11 from each individual), and therefore it is unlikely to really be a false positive. Finally, ebwt2clust finds more non-isolated SNPs than DiscoSnp++.

With parameter $b=1$, DiscoSnp++ exhibits a sensitivity and precision similar to those of ebwt2clust ’s output filtered on $cov=8$. The major difference between the two tools consists in the higher number of non-isolated SNPs found by ebwt2clust ($52.47\%$ versus $45.57\%$ of DiscoSnp++).

To conclude, DiscoSnp++ with parameter $b=2$ exhibits a sensitivity similar to that of ebwt2clust ’s output filtered on $cov=6$. In this case, ebwt2clust has a higher precision with respect to the ground truth, but it also outputs a smaller absolute number of SNPs. Again, ebwt2clust finds more non-isolated SNPs than DiscoSnp++.

## Conclusions and further works

We introduced a positional clustering framework for the characterization of breakpoints of genomic sequences in their eBWT, paving the way to several possible applications in assembly-free and reference-free analysis of NGS data. The experiments proved the feasibility and potential of our approach.

We note that our analysis automatically adapts to the case where also indels are present in the reads (i.e. not just substitutions, but possibly also insertions and deletions). To see why this holds true, note that our analysis only looks at the *first* base that changes between the two individuals in a cluster containing similar read suffixes. Since we do not look at the following bases, indels behave exactly like SNPs in Theorems 3.2 and 3.3: indels between the two individuals will produce clusters containing two distinct letters (i.e. we capture the last letter of the indel in one individual, which by definition differs from the corresponding letter in the other individual). By extracting also the left-context preceding the ebwt cluster and performing a local alignment, one can finally discover the event type (SNP or INDEL). We plan to implement this feature in a future extension of our tool.

Further work will focus on improving the prediction in highly repeated genome regions and using our framework to perform haplotyping, correcting sequencing errors, detecting alternative splicing events in RNA-Seq data, and performing sequence assembly. We also plan to improve the efficiency of our pipeline by replacing the GSA/LCP arrays—which at the moment force our pre-processing step to be performed in external memory—by an FM-index. By switching to internal-memory compressed data structures, we expect to speed up both eBWT computation and ebwt2snp analysis. Finally, since the scan of the eBWT and the LCP that detects the cluster is clearly a local search, we plan to implement a parallelisation of our SNPs calling tool expecting a much lower running time.

## Footnotes

1 A *bubble* in a graph is a pair of disjoint paths sharing the same source node and target node.

2 For simplicity of exposition, here we assume that all the reads have the same length *r*. With little more effort, it can be shown that our results hold even when *r* is the average read length.

3 https://github.com/giovannarosone/BCR_LCP_GSA.

4 https://github.com/felipelouza/egsa.

5 https://github.com/felipelouza/sacak-lcp.

6 .vcf stands for *Variant Call Format*: the standard text format for storing genome sequence variations with meta-information about position in the reference genome.

7 http://bio-bwa.sourceforge.net/.

8 ftp.1000genomes.ebi.ac.uk//vol1/ftp/technical/reference/phase2_reference_assembly_sequence/hs37d5.fa.gz.

9 ftp.1000genomes.ebi.ac.uk/vol1/ftp/release/20130502/.

10 https://github.com/jstjohn/SimSeq/blob/master/examples/hiseq_mito_default_bwa_mapping_mq10_1.txt.
